# *Ageratum enation virus*—A Begomovirus of Weeds with the Potential to Infect Crops

**DOI:** 10.3390/v7020647

**Published:** 2015-02-10

**Authors:** Muhammad Tahir, Imran Amin, Muhammad Saleem Haider, Shahid Mansoor, Rob W. Briddon

**Affiliations:** 1 Plant Biotechnology Department, Atta-ur-Rahman School of Applied Biosciences, National University of Sciences and Technology, Sector H-12, Islamabad 44000, Pakistan; 2 Agricultural Biotechnology Division, National Institute for Biotechnology and Genetic Engineering, Jhang Road, Faisalabad 38000, Pakistan; E-Mails: imranamin1@yahoo.com (I.A.); shahidmansoor7@gmail.com (S.M.); rob.briddon@gmail.com (R.W.B.); 3 School of Biological Sciences, University of the Punjab, New Campus, Lahore 54590, Pakistan; E-Mail: haider65us@yahoo.com

**Keywords:** geminivirus, begomovirus, betasatellite, ssDNA virus

## Abstract

Samples of two *Ageratum conyzoides*, one *Sonchus oleraceu*s and one turnip (*Brassica rapa* var. rapa) exhibiting virus-like symptoms were collected from Pakistan and Nepal. Full-length begomovirus clones were obtained from the four plant samples and betasatellite clones from three of these. The begomovirus sequences were shown to be isolates of *Ageratum enation virus* (AEV) with greater than 89.1% nucleotide sequence identity to the 26 AEV sequences available in the databases. The three betasatellite sequences were shown to be isolates of Ageratum yellow leaf curl betasatellite (AYLCB) with greater than 90% identity to the 18 AYLCB sequences available in the databases. The AEV sequences were shown to fall into two distinct strains, for which the names Nepal (consisting of isolates from Nepal, India, and Pakistan—including the isolates identified here) and India (isolates occurring only in India) strains are proposed. For the clones obtained from two AEV isolates, with their AYLCB, infectivity was shown by *Agrobacterium*-mediated inoculation to *Nicotiana benthamiana*, *N. tabacum*, *Solanum lycopersicon* and *A. conyzoides*. *N. benthamiana* plants infected with AEV alone or betasatellite alone showed no symptoms. *N. benthamiana* plants infected with AEV with its associated betasatellite showed leaf curl symptoms. The findings show that AEV is predominantly a virus of weeds that has the capacity to infect crops. AYLCB appears to be the common partner betasatellite of AEV and is associated with diseases with a range of very different symptoms in the same plant species. The inability to satisfy Koch’s postulates with the cloned components of isolate SOL in *A. conyzoides* suggests that the etiology may be more complex than a single virus with a single betasatellite.

## 1. Introduction

Viruses of the family *Geminiviridae* are important pathogens of cultivated plants throughout the warmer parts of the World and are also increasingly spreading into more temperate areas. Mainly a problem in developing nations of the tropics, some of the viruses also cause significant problems in more developed countries. A good example is *Tomato yellow leaf curl virus* (TYLCV). TYLCV has its origins in the eastern Mediterranean/Middle East but has in the last 20 years spread across southern Europe, North Africa, the Caribbean, parts of the Americas, China, Japan and Australia [1]. Much of our knowledge of the diversity of geminiviruses comes from the study of viruses found in cultivated plants [2]. Only fairly recently has the focus of investigation also included weed species. These efforts have shown that the diversity of geminiviruses is extremely wide and that weeds may harbour known, agriculturally important virus species, as well as previously unidentified virus species, which could potentially affect cultivated plants in the future [3,4].

Geminiviruses have genomes consisting of circular, single-stranded (ss) DNA that are encapsidated in characteristic twinned, quasi-icosahedral particles. Viruses in the family are assigned to one of seven genera based on their host range, genome arrangement and insect vector [5,6]. The majority of geminiviruses, as well as economically most important, are in the genus *Begomovirus*. Begomoviruses are transmitted exclusively by the whitefly *Bemisia tabaci* to dicotyledonous hosts. The majority of begomoviruses native to the New World have genomes consisting of two ssDNA components of approximately equal size (~2800 nt) that are designated DNA A and DNA B, both of which are required for systemic infection of plants. The DNA A component encodes all viral functions required for viral DNA replication, control of gene expression and transmission between plants whereas the DNA B component encodes two proteins required for inter- and intracellular movement in host plants [7].

Although some bipartite begomoviruses occur in the Old World, most are monopartite, their genomes being a homolog of the DNA A component of bipartite viruses. A small number of these viruses are truly monopartite, but the majority instead associate with a group of ssDNA satellites known collectively as betasatellites [8]. Betasatellites are typically half the size of their helper begomoviruses (~1350 nt) and are required by their helper viruses to infect the plants species from which they were isolated but require the helper virus for replication and spread in plants as well as transmission between plants [9]. All betasatellites identified thus far have a highly conserved structure consisting of a single open reading frame (known as βC1), a region of sequence rich in adenine (A-rich) and a sequence highly conserved between all isolates known as the satellite conserved region (SCR). The SCR contains a predicted hairpin structure with the loop containing the nonanucleotide sequence TAATATTAC with similarity to the origin of virion-strand DNA replication of geminiviruses [10].

The genomes of monopartite begomoviruses encode six genes. The two in the virion-sense encode the coat protein (CP), involved in virus movement within and between plants, and the V2 protein, which is involved in virus movement in plants as well as, for some species, in overcoming host defenses triggered by double-stranded RNA (known as RNA interference [RNAi]) [7,11]. The complementary-sense genes encode the transcriptional activator protein (TrAP; a transcription factor that, for some begomoviruses, up-regulates expression of the virion-sense genes, modulates host gene expression and may be involved in overcoming RNAi), the replication enhancer protein (REn; that is involved in providing a cellular environment suitable for virus replication [12]), the C4 protein (that may be involved in overcoming RNAi and may be a pathogenicity determinant [13,14]) and the replication associated protein (Rep).

Rep is a rolling circle replication (RCR) initiator protein that interferes with host cell-cycle and is the only virus encoded protein required for virus replication [15]). To initiate RCR Rep recognizes and binds short repeated sequences (known as “iterons”), adjacent to the TATA box of the complementary-sense promoter within the non-coding intergenic region (IR) of the virus genome [16,17]. The amino acid sequences of Rep predicted to interact with iterons are known as the “iteron related domain” (IRD) [18].

The analysis presented here concerns a virus, *Ageratum enation virus* (AEV), first identified in Nepal in the late 1990s. We show that AEV has two distinct strains, and to occur across a large geographic area. Although initially isolated from weeds, we show it is also now crossing into cultivated species and could become a more significant problem for agriculture in the future.

## 2. Materials and Methods

### 2.1. DNA Extraction, PCR Amplification and Cloning

Total nucleic acids were extracted from leaf samples as previously described [19]. For isolates SOL and ACL primer pair WTGF/WTGR [20] were used to PCR amplify an approximately 1500 bp fragment spanning all of the intergenic region and most of the Rep gene (data not shown). A specific pair of abutting primers BGAF/BGAR [21] containing a unique restriction enzyme (*Apa*1) site, to PCR-amplify the complete begomovirus genome. The full-length begomovirus from isolate ABF was PCR-amplified with primer pair BF/BR [22]. The full-length genome of the begomovirus from isolate ACN was PCR-amplified using abutting primers AAGCTTTGATGAGTTCCGCTG/AAGCTTCTCAAGCAGAGAATGGCG containing a unique *Hind*III restriction site designed to a partial sequence obtained with the universal begomovirus primers described previously [23]. Betasatellites were amplified with universal primers beta01/beta02 [24]. Potentially full-length amplification products for were cloned using either the InsT/A clone PCR product cloning kit (Fermentas) or the pGEM-T Easy kit (Promega), as recommended by the manufacturers.

### 2.2. Sequencing and Sequence Analysis

The complete nucleotide sequences of potentially full-length clones were determined by dideoxynucleotide chain-termination sequencing using Genome Lab Dye Terminator Cycle Sequencing kits (Beckman Coulter, Nyon, Switzerland) with reaction products run on a Beckman Coulter automated sequencer (CEQ 8000) or commercially (Macrogen [Seoul, Korea] or Lark Technologies [Takeley, UK]). Sequence information was stored, assembled and analyzed using the Lasergene sequence analysis package (DNAStar Inc., Madison, WI, USA).

Phylogenetic analyses were conducted on matrices of aligned sequences using the neighbor-joining and bootstrap options of Phylip (v. 3.5c) running on an IBM compatible personal computer. Sequence alignments were produced using CLUSTAL X [25]. Phylogenetic dendrograms were viewed, manipulated and printed using Treeview [26]. All sequence alignments to assess the taxonomic status of sequences (viruses/betasatellites) used the Clustal V algorithm (as implemented in MegAlign, Lasergene package), as recommended by the *Geminiviridae* Study Group of the International Committee on Taxonomy of Viruses [27].

### 2.3. Constructs for Infectivity

Partial direct repeat constructs of the begomoviruses and betasatellites cloned from isolates SOL and ACL were produce for the analysis of infectivity. For the begomovirus clones an *Apa*I*-Xba*I fragment of ~1.6 kb, containing the entire intergenic region, was released from the full-length clone and ligated into the binary vector pGreen0029 [28]. The full-length begomovirus insert, released with *Apa*I, was then ligated into the *Apa*I site of the pGreen vector containing the 1.6 kb fragment to yield a 1.6-mer partial tandem repeat construct. A similar strategy, but using restriction endonucleases *Kpn*I and *Cla*I, was followed to produce partial repeat constructs of the betasatellites.

### 2.4. Agrobacterium-Mediated Inoculation

Plants for inoculation were grown in a glasshouse with a 16 h light/8 h dark cycle at 28 °C. The binary vector constructs were electroporated into *Agrobacterium tumefaciens* strain GV3101. *Agrobacterium* cultures for inoculation were grown at 28 °C for 48 h to an optical density at 600 nm of 0.6 with antibiotic selection. The bacterial cells were pelleted (5000 g for 15 min at 4 °C) and resuspended in 10 mM MgCl_2_ and 150 µg/mL of acetosyringone. Cells were incubated on ice for 3 h and then inoculated into plants as previously described [29].

## 3. Results

### 3.1. Cloning, Sequencing and Sequence Analysis of Begomoviruses

**Figure 1:**
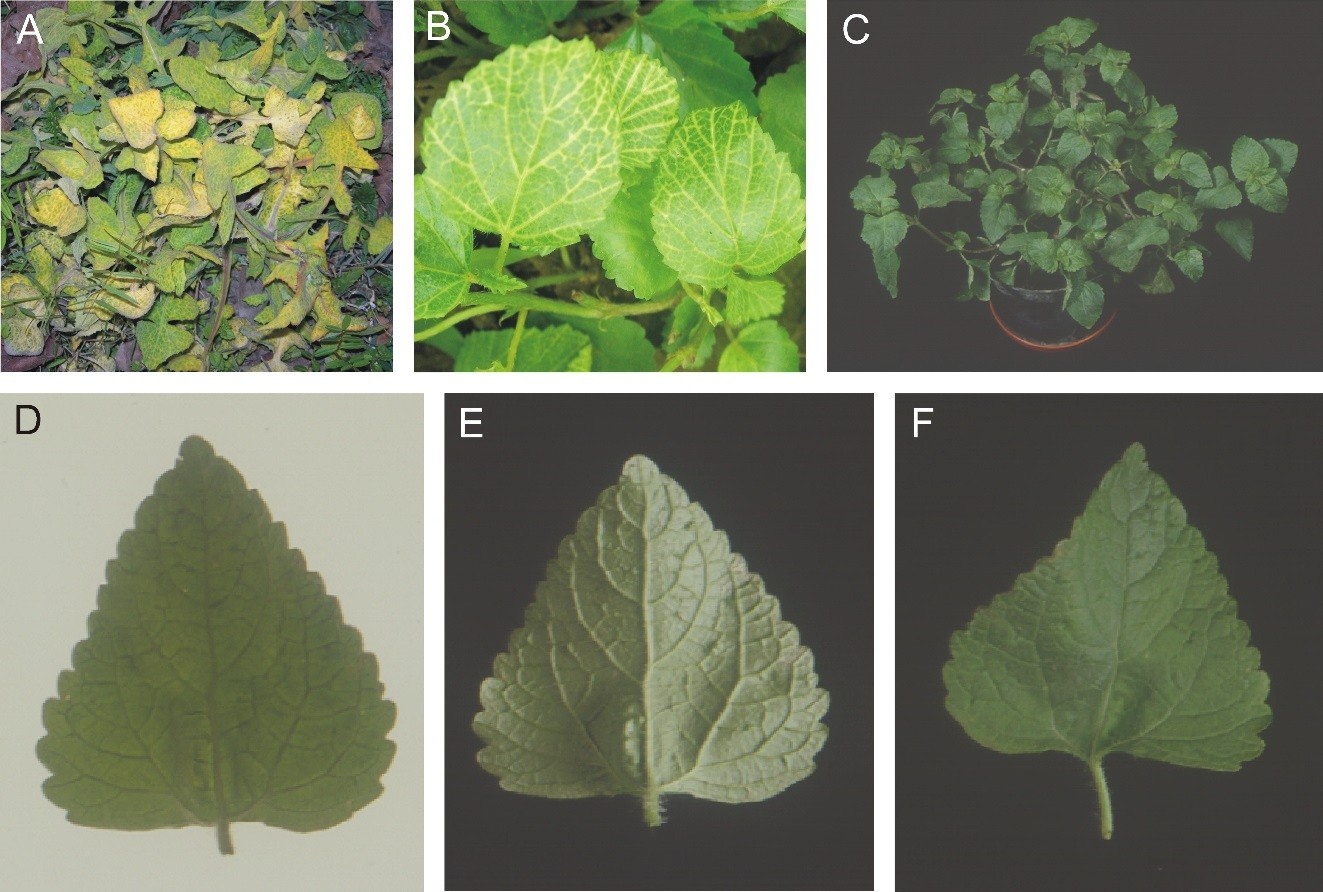
Symptoms exhibited by the plants from which AEV clones were obtained. *Sonchus oleraceous* with yellow veins (**A**) *Ageratum conyzoides* with yellow veins (**B**), *A. conyzoides* from Nepal (**C**). A leaf from the *A. conyzoides* plant from Nepal is photographed under transmitted light to highlight the vein darkening (**D**) and under reflected light on the underside (**E**) and upper side (**F**) to highlight the dimple structures.

Leaf samples of field infected weeds *Ageratum conyzoides* (isolate ACL) and *Sonchus oleraceous* (SOL), showing yellow vein symptoms (Figure 1, panels A and B), were collected from Lahore, Pakistan, in 2006 and 2005, respectively. A leaf sample of turnip (*Brassica rapa* var. rapa), exhibiting foliar vein yellowing, was collected from Faisalabad, Pakistan during 2006 (isolate ABF). Additionally a leaf sample originating from an *A. conyzoides* plant, collected in Nepal during the 1990s (isolate ACN), showing a novel symptom was included. This plant appeared superficially healthy (Figure 1, panel C). However, closer inspection of leaves under transmitted light showed vein darkening (Figure 1, panel D) and, on the lower leaf surface, showed raised veins and structures on veins resembling enations (Figure 1, panel E) which on the upper leaf surface were depressions (Figure 1, panel F)—we shall refer to this novel symptom as “dimples”.

The complete nucleotide sequences of single begomovirus clones from each of the four isolates were determined. The sequences were determined to be 2750 bp, 2749 bp, 2747 bp and 2746 bp, respectively, in length and are available in the databases under accession numbers given in Table 1. Analyses of the sequences shows them, in all respects, to be typical of the genomes of monopartite and DNA A components of bipartite begomoviruses encoding two genes (for the CP and V2 protein) in the virion-sense and four genes (for the Rep, TrAP, REn and C4 protein) in the complementary-sense. The positions and coding capacity of the predicted genes are given in Table 1. Between the virion- and complementary-sense genes lies a non-coding intergenic region which contains a predicted hairpin structure with, within the loop, the conserved (between all geminiviruses) nonanucleotide sequence (TAATATTAC) which forms part of the origin or virion-strand DNA replication.

**Table 1:** Origins of the virus isolates and features of the begomovirus and betasatellite clones obtained.

Isolate	Origin (year)	Plant Species (Symptoms *)	Component	Accession No.	Size (bp)	ORF	Start/Stop Codon (Nucleotide Coordinates)	Predicted Size (no. of Amino Acids)	Predicted Molecular Weight (kDa)
SOL	Lahore, Pakistan (2005)	*Sonchus oleraceus* (YV)	begomovirus	AM261836	2750	V2	135/482	115	13.3
						CP	295/1065	256	29.7
						Rep	2599/1514	361	40.7
						TrAP	1611/1180	143	16.2
						REn	1466/1062	134	15.9
						C4	2442/2185	85	9.3
			betasatellite	AM412239	1368	βC1	605/189	138	16.1
ACL	Lahore, Pakistan (2006)	*Ageratum conyzoides* (YV)	begomovirus	AM698011	2749	V2	134/481	115	13.3
						CP	294/1064	256	29.6
						Rep	2598/1513	361	40.8
						TrAP	1610/1206	134	15.0
						REn	1465/1061	134	15.9
						C4	2441/2184	85	9.4
			betasatellite	AM698010	1355	βC1	596/180	138	16.1
ACN	Nepal (2001)	*Ageratum conyzoides* (LC, EN)	begomovirus	AJ437618	2746	V2	134/481	115	13.3
						CP	294/1064	256	29.5
						Rep	2597/1512	361	40.6
						TrAP	1610/1206	134	15.1
						REn	1465/1061	134	15.9
						C4	2441/2184	85	9.2
			betasatellite	-	-	-	-	-	-
ABF	Faisalabad, Pakistan (2006)	*Brassica rapa* var. rapa (YV)	begomovirus	AM701770	2747	V2	134/481	115	13.3
						CP	294/1064	256	29.6
						Rep	2598/1513	361	40.8
						TrAP	1610/1206	134	15.2
						REn	1465/1061	134	15.8
						C4	2441/2184	85	9.4
			betasatellite	AM701771	1359	βC1	596/180	138	16.1

* Symptoms exhibited by plants from which the virus/betasatellite clones were isolated. Symptoms are denoted as yellow vein (YV), leaf curl (LC), enations (E).

Despite being isolated from widely different regions (Nepal and Pakistan) and with a 10 year gap, the four sequences show high levels of sequence conservation (between 95.2%–98.7% nucleotide sequence identity). Based on the 89% nucleotide sequence identity demarcation threshold for identification of begomovirus species, this indicates that all four clones are isolates of a single species [27,30].

### 3.2. Comparison of the Sequences Obtained Here with Sequences Available in the Databases

The AEV clone obtained from Nepal was obtained some time ago and was at the time recognized as a distinct species within the genus *Begomovirus* and given the name *Ageratum enation virus* [30]. The sequence of AEV-Nepal thus represents the type isolate of this begomovirus species. The other three sequences, with greater than 89% nucleotide sequence identity to AEV-Nepal, thus are variants of AEV. A Blast search of the GenBank nucleotide sequence database with the four AEV sequences presented here identified 26 additional sequences originating from India with high sequence identity (>89%): GQ268327 isolated from *Trichosanthes dioica* (unpublished), EU867513 isolated from *Amaranthus cruentus* (unpublished), KC795968 and KC818421 isolated from tomato (unpublished), JX436472 isolated from tomato (unpublished), HE861940 isolated from soybean (unpublished), JQ911765 and HM149260 isolated from *Papaver somniferum* (unpublished), JX436473 isolated from Fenugreek (unpublished), JQ911767, KJ488990 and KJ488991 isolated from *Ageratum* sp. (unpublished), JF728865 and JF728867 (isolated from carrot [31]), JF728860-JF728864 and JF728866 isolated from *A. conyzoides* [31], JF682242 isolated from *Amaranthus* (unpublished), FN794201 isolated from *Crassocephalum crepidioides* [32], FN543099 isolated from *Zinnia elegans* [33], FJ177031 isolated from *Cleome gynandra* [34] and FN794198 isolated from *A. conyzoides* [32]. Some of these sequences have previously been reported as isolates of AEV. Overall these 30 sequences (including the 4 identified here) show between 88.9% and 100% nucleotide sequence identity (Table S1), with the highest levels of identity to isolates of *Tobacco curly shoot virus* (TbCSV). This confirms the 30 sequences as isolates of a distinct begomovirus species, which has previously been named AEV [30].

A closer analysis of the AEV sequences shows them to fall into three groups. The first group consists of all the AEV sequences characterized here, as well as GQ268327, EU867513, KC818421, KC795968, and FJ177031. These nine sequences show between 93.9% and 100% nucleotide sequence identity (Table S1). The sequences in the second group, consisting of FN543099, FN794198, FN794201, JF728860-64, JF728866, JX436472, JX436473, JQ911765, JF682242, JQ911767, HM149260, KJ488991, KJ488990 and HE861940 show between 94.6% and 100% identity. Between the two groups the identity levels vary between 90.6%. and 92.9%. Based on the presently applicable demarcation threshold for strains within a species (93%; [27]), this would indicate that the two groups represent strains of AEV, for which we propose the names “Nepal” and “India”, respectively. The third group of sequences (JF728865 and JF728867) show relatively high sequence identities to both the Nepal (group 1—91.8% to 98.1% identity) and the India (group 2—94.3% to 99.2% identity) strain AEV sequences, making it difficult to assign them to a strain.

Interestingly the four AEV sequences have Rep IRD sequences predicted to be either FKIN (AM261836, AM698011, AM701770) or LKIN (AJ437618). The other three Nepal strain isolates also have LKIN. In contrast, all India strain isolates have an IRD with the predicted sequence FQIY. The corresponding iteron sequences are GGT/AGT for all Nepal strain isolates and either GGTG/AC/A, or possibly GTACT, for all India strain isolates and both problematic isolates. The alignment of all AEV sequences suggested that the differences between the Nepal and India strain isolates may be due to recombination across the origin of replication, possibly with *Papaya leaf crumple virus* (PaLCrV) as the donor of the ori for the India strain (results not shown). PaLCrV occurs in India and has IRD and iteron sequences identical to those of India strain AEV sequences.

**Figure 2:**
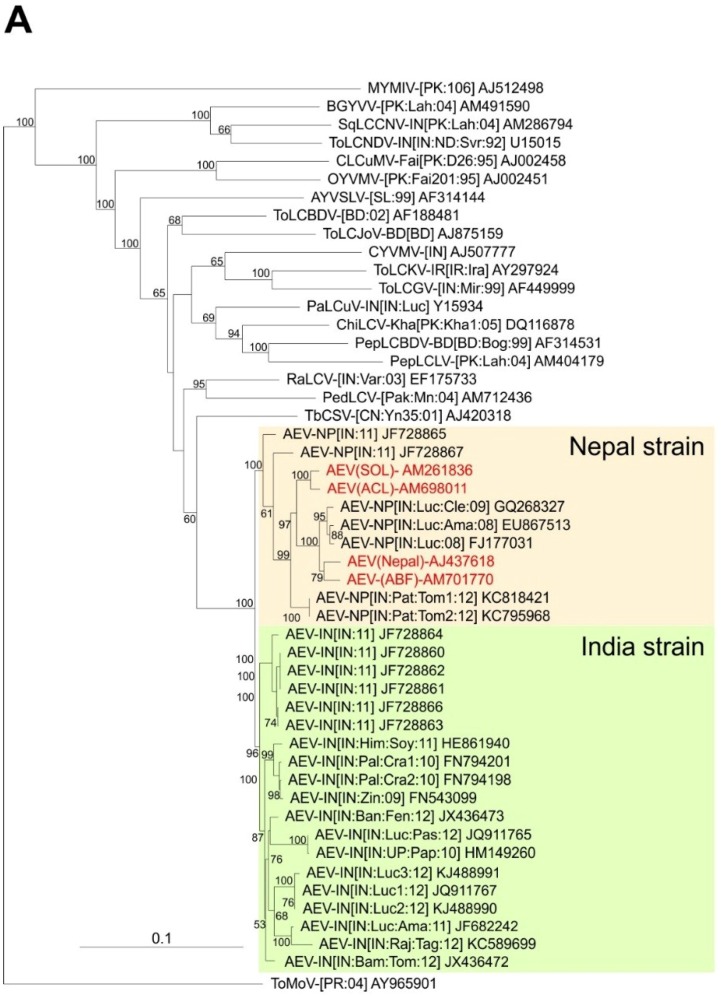

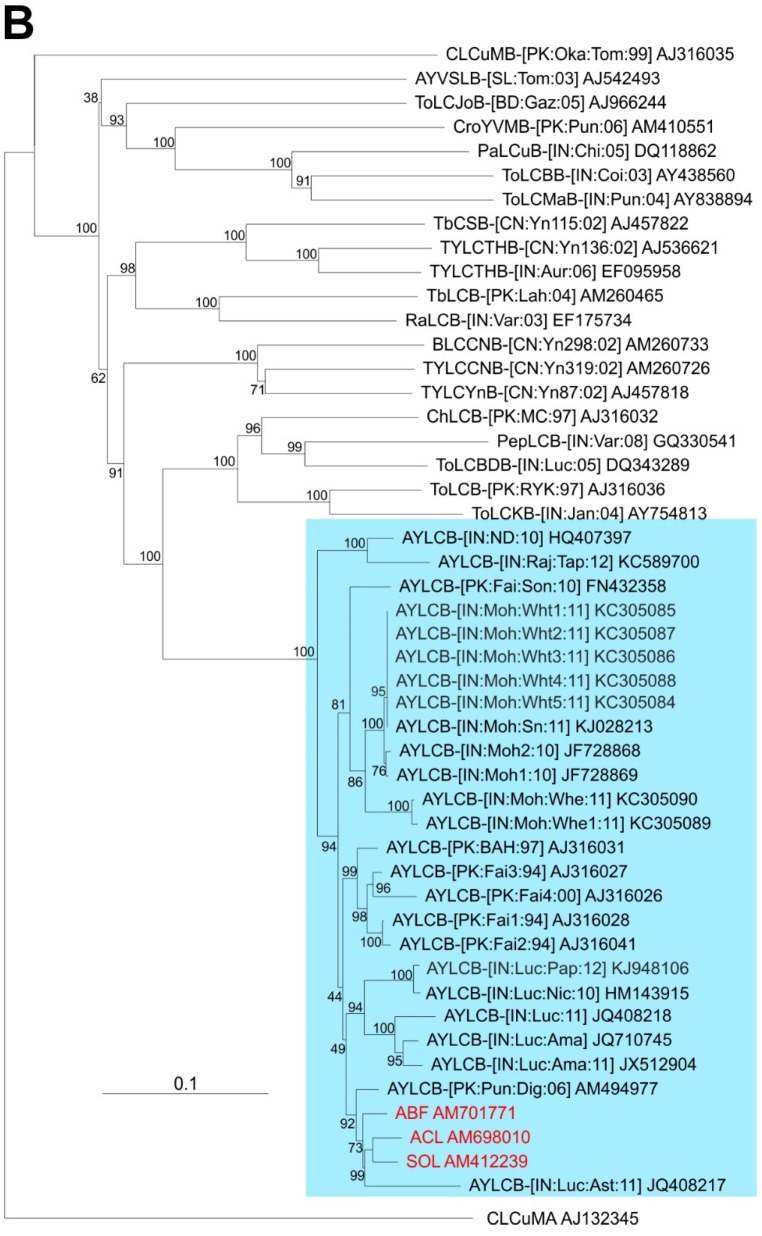
Phylogenetic analyses of begomovirus and betasatellite sequences. Phylogenetic dendrograms based upon alignments of the complete nucleotide sequences of the genomes (or DNA A genomic components of) begomoviruses (**A**) and betasatellites (**B**) identified here with selected sequences available in the databases. The Neighbour-joining method was used for construction of the phylogenetic dendrograms. Horizontal distances are proportional to mutation distances whereas vertical distances are arbitrary. The numbers at each branch indicate percentage bootstrap confidence scores (1000 replicates). The begomovirus acronyms used are *Ageratum enation virus* (AEV), *Ageratum yellow vein Sri Lanka virus* (AYVSLV), *Bitter gourd yellow vein virus* (BGYVV), *Chili leaf curl virus* (ChiLCV), *Cotton leaf curl Multan virus* (CLCuMV), *Croton yellow vein mosaic virus* (CYVMV), *Okra yellow vein mosaic virus* (OYVMV), *Mungbean yellow mosaic India virus* (MYMIV), *Papaya leaf curl virus* (PaLCuV), *Pedilanthus leaf curl virus* (PedLCV), *Pepper leaf curl Bangladesh virus* (PepLCBDV), *Pepper leaf curl Lahore virus* (PepLCLV), *Radish leaf curl virus* (RaLCV), *Squash leaf curl China virus* (SLCCNV), *Tobacco curly shoot virus* (TbCSV), *Tomato leaf curl Bangladesh virus* (ToLCBDV), *Tomato leaf curl Gujarat virus* (ToLCGV), *Tomato leaf curl Joydebpur virus* (ToLCJoV), *Tomato leaf curl Karnataka virus* (ToLCKV) and *Tomato leaf curl New Delhi virus* (ToLCNDV). Isolate descriptors are as given in [27]. The tree was rooted on the sequence of the DNA A component of *Tomato mottle virus* (ToMoV) as an outgroup. The AEV isolates and the two strains of AEV (India and Nepal) are indicated on the right of the tree. The betasatellite acronyms used are Ageratum yellow leaf curl betasatellite (AYLCB), Ageratum yellow vein Sri Lanka betasatellite (AYVSLB), Bean leaf curl China betasatellite (BLCCNB), Chili leaf curl betasatellite (ChLCB), Cotton leaf curl Multan betasatellite (CLCuMB), Tomato leaf curl Joydebpur betasatellite (ToLCJoB), Croton yellow vein mosaic betasatellite (CroYVMB), Papaya leaf curl betasatellite (PaLCuB), Pepper leaf curl betasatellite (PepLCB), Radish leaf curl betasatellite (RaLCB), Tobacco curly shoot betasatellite (TbCSB), Tobacco leaf curl betasatellite (TbLCB), Tomato leaf curl Bangalore betasatellite (ToLCBB), Tomato leaf curl Bangladesh betasatellite (ToLCBDB), Tomato leaf curl Karnataka betasatellite (ToLCKB), Tomato leaf curl Maharashtra betasatellite (ToLCMaB), Tomato leaf curl betasatellite (ToLCB), Tomato yellow leaf curl China betasatellite (TYLCCNB), Tomato yellow leaf curl Thailand betasatellite (TYLCTHB) and Tomato yellow leaf curl Yunan betasatellite (TYLCYnB). Isolate descriptors are as given in [8]. The tree was rooted on the sequence of Cotton leaf curl Multan alphasatellite (CLCuMA) as outgroup. For both trees the sequences obtained here are highlighted in red text and the database accession numbers of isolates are given.

A phylogenetic dendrogram based upon an alignment of the four sequences obtained here, the 26 other AEV sequences available in the databases and selected other begomovirus sequences available in the databases is shown in Figure 2A. This shows the 30 AEV sequences to form a group distinct from all other sequences in the tree but to be most closely related to TbCSV. The division of AEV sequences into two distinct groups, for which we have proposed the names Nepal and India strains, are well supported by bootstrapping. The two problematic AEV isolates (JF728865 and JF728867) group with, but are distinct from and basal to the Nepal strain isolates, despite having overall more sequence identity to isolates of the India strain. JF728867 has a Nepal strain IRD (FKIN), whereas JF728865 has an Indian strain IRD (FQIY). It is for this reason that we proposed, at least provisionally, to include these two isolates under the Nepal strain, awaiting the availability of further sequences and analyses.

### 3.3. Cloning, Sequencing and Sequence Analysis of Betasatellites

Three full-length betasatellite clones were obtained. Unfortunately the satellite for isolate ACN was not obtained for comparison, although the presence of a betasatellite with this isolate has been shown previously [35]. The three sequences have a length typical of betasatellites (between 1355 to 1368 bp, Table 1), being approximately half the size of their helper begomoviruses. Analysis of the sequences show them to have an arrangement typical of this class of satellites consisting of a single open reading frame in the complementary-sense encoding the βC1 protein (Table 1), a region of sequence rich in adenine and a sequence highly conserved between all betasatellites—the satellite conserved region [35].

Comparison of the three betasatellite sequences obtained here to sequences available in the databases shows them to be most similar to Ageratum yellow leaf curl betasatellite (AYLCB; with between 90.6% and 99.8% nucleotide sequence identity to the 18 other sequences available in the databases). Six defective AYLCB are available in the databases (JQ408217, KC305086-90) ranging in size from 891 bp to 1270 bp, which are included in the analysis but not included in the calculated identity values. To all other betasatellite sequences available in the databases the percentage nucleotide sequence identity was less than 62% (not shown) with the highest sequence identity levels (57.5%–61.3%) to an isolate of Tomato leaf curl Bangladesh betasatellite (AJ542489).

A phylogenetic dendrogram, based upon an alignment of the full-length sequences of the 3 betasatellites obtained here, all available AYLCB sequences in the databases and selected other betasatellite sequences from the databases is shown in Figure 2B. This shows AYLCB to form a clade with Chili leaf curl betasatellite, Pepper leaf curl betasatellite, Tomato leaf curl Bangladesh betasatellite, Tomato leaf curl betasatellite and Tomato leaf curl Karnataka betasatellite—a grouping well supported by bootstrapping. It is interesting to note that all AYLCB isolates from Pakistan, with the exception of FN432358, are distinct from those originating from India. This suggests that AYLCB in these two areas are evolving independently. Possibly FN432358, an “Indian” isolate of AYLCB occurring in Pakistan, indicates that infrequent exchanges between India and Pakistan take place. Also, the Pakistan AYLCB isolates characterized prior to 2000 are distinct from those characterized more recently, which may indicate temporal changes that have occurred in this betasatellite species.

### 3.4. Analysis of the Infectivity of AEV and Betasatellite Clones

**Figure 3:**
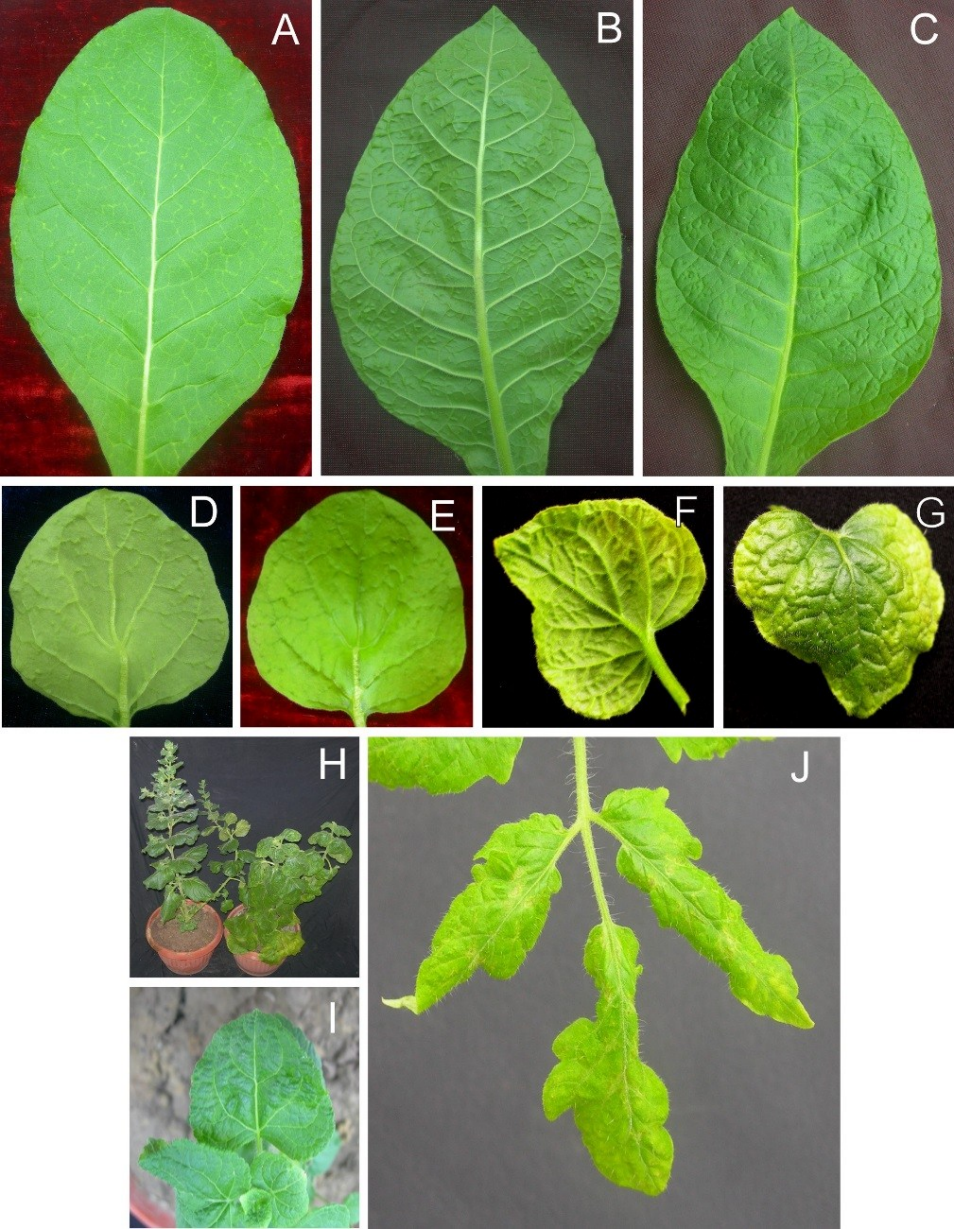
Symptoms of plants infected with AEV and AYLCB clones obtained from isolate SOL. *Nicotiana tabacum* plants infected with AEV^SOL^/AYLCB^SOL^ initially (15 dpi) showed mild vein yellowing on leaves developing subsequent to inoculation (**A**) which developed into what looked, on the underside of the leaf, like vein swelling (**B**) but on the upper leaf surface was evidently depression of the veins (**C**) at 20 dpi. Infected *N. benthamiana* initially (12 dpi) exhibited enations on the veins on the undersides of leaves (**D**) that on the upper leaf surface were seen to be depressions (dimples; **E**). In older *N. benthamiana* infections (30 dpi) leaves showed extensive chlorosis with raised veins on the lower surface (**F**) and sunken veins on the upper surface (**G**) giving a crumpled appearance. A comparison of a healthy, non-inoculated *N. benthamiana* plant (left) and an AEV^SOL^/AYLCB^SOL^ infected plant at 30 dpi is shown in panel (**H)**. The infected plant showed some chlorosis and overall downward curved leaves. The height of the plant was not greatly affected. *Ageratum conyzoides* plants infected with AEV^SOL^/AYLCB^S^°^L^ showed mild leaf crumpling but no vein yellowing (**I**). Infected *Solanum lycopersicon* plants showed chlorosis of leaves developing after inoculation and vein necrosis (**J**).

Partial repeat constructs of the AEV and betasatellite clones isolated from *S. oleraceus* (isolate SOL) and *A. conyzoides* (isolate ACL) were produced for *Agrobacterium*-mediated inoculation of plants. The results of the infectivity studies are summarized in Table 2. Inoculation of *N. benthamiana* with the AEV and AYLCB clones isolated from *S. oleraceus* (AEV^SOL^ and AYLCB^SOL^) led to the appearance of symptoms consisting of vein thickening and dimples (structures that, on the underside of the leaf, resembled enations but on the upper leaf surface were depressions; Figure 3, panels D and E) on veins at 12 days post inoculation (dpi) of leaves developing subsequent to inoculation. At approximately 18 to 20 dpi the dimples were more pronounced and were associated with severe leaf curling, followed by foliar chlorosis at 30 dpi with the veins being raised on the lower surface and depressed on the upper surface (Figure 3, panels F and G). Older plants were stunted, in comparison to healthy *N. benthamiana* plants, with leaves showing a distinct downward curling and chlorosis (panel H).

For *N. tabacum*, inoculation with AEV^SOL^ and AYLCB^SOL^ initially resulted in mild vein yellowing (at approx. 15 dpi; Figure 3, panel A) followed by sunken veins (on the upper leaf surface; appearing as swollen vein on the lower surface)) and dimple structures, similar to those seen in *N. benthamiana*, at 20 dpi (Figure 3, panels B and C). However, *N. tabacum* did not exhibit the leaf curling and chlorosis evident in *N. benthamiana.*

*A. conyzoides* plants infected with AEV^SOL^/AYLCB^SOL^ initially showed mild leaf crumpling at 12 dpi. At approx. 18 dpi the leaves developing subsequent to inoculation showed crumpling and some leaf curling (Figure 3, panel I).

For infected *S. lycopersicon* plants inoculated with AEV^SOL^ and AYLCB^SOL^ the leaves developing subsequent to inoculation initially showed vein yellowing at 15 dpi. By 21 dpi leaves were narrow, rolled at the edges and showed a patchy necrosis developing from the veins (Figure 3, panel J).

**Figure 4:**
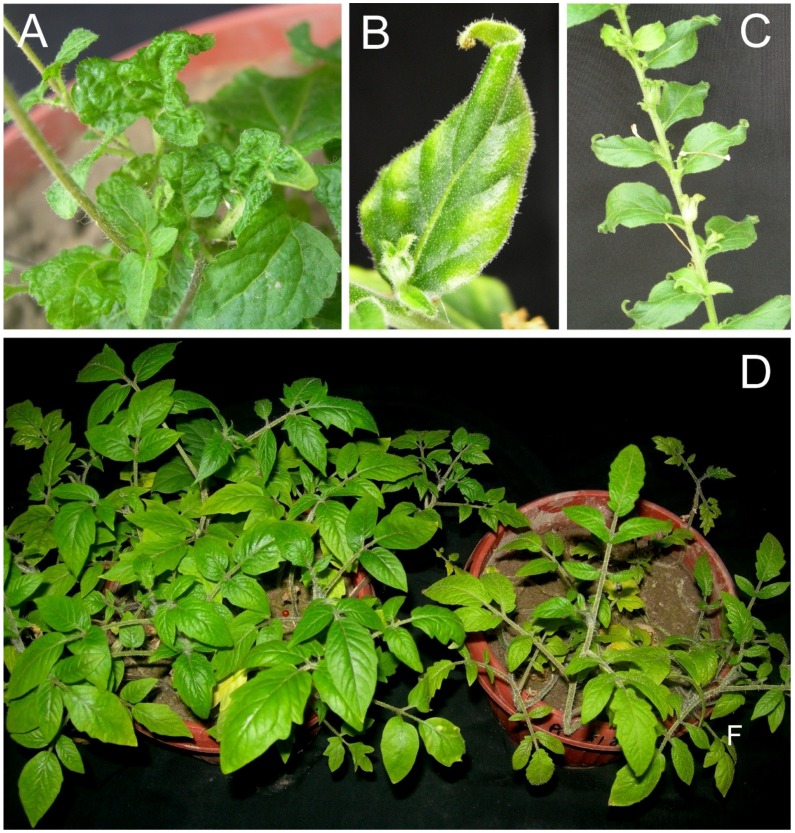
Symptoms of plants infected with AEV and AYLCB clones obtained from isolate ACL. *Ageratum conyzoides* plants infected with AEV^ACL^/AYLCB^ACL^ with severely distorted leaves but no evidence of vein yellowing (**A**). The leaves of *N. benthamiana* plants developing subsequent to inoculation were very narrow with sharp, curled tips and some chlorosis ((**B**) and some lateral curling (**C**)). Symptoms in *Solanum lycopersicon* were mild with a reduction in leaf size, mild chlorosis and plan stunting ((**D**), right) in comparisons to a healthy, non-inoculated plant (left). Plants were photographed at approx. 30 dpi.

Inoculation of *N. benthamiana* with the clones obtained from isolate ACL (AEV^ACL^ and AYLCB^ACL^) led to the first symptoms appearing within 12 dpi, consisting of narrow leaves with a pointed apex with some foliar chlorosis for leaves developing subsequent to inoculation. As plants developed, the leaves remained small and chlorotic with some twisting (Figure 4, panels B and C). Inoculated *A. conyzoides* plants similarly showed the first symptoms within 12 dpi consisting of crumpling in leaves developing after inoculation. Subsequently plants remained severely stunted, with severe crumpling and deformation of leaves (Figure 4, panel A). For tomato (*S. lycopersicon*) plants inoculated with AEV^ACL^ and AYLCB^ACL^ the symptoms were not very distinct with older plants showing stunting, reduced leaf size and a mild foliar chlorosis (Figure 4, panel D). On some older leaves a mild veinal necrosis developed, but this was not as pronounced as for tomato plants inoculated with AEV^SOL^/AYLCB^SOL^.

**Table 2:** Infectivity of AEV, isolates SOL and ACL, with their AYLCB by *Agrobacterium*-mediated inoculation.

Inoculum	Plant Species	Infectivity (Plants Infected/Plants Inoculated)	Latent Period ^#^ (days)	PCR Detection		Symptoms *
				Virus	Betasatellite	
AEV^SOL^ and AYLCB^SOL^	*N. benthamiana*	60/60	12–18	+	+	D, LC, C
	*N. tabacum*	20/20	12–18	+	+	VY, D
	*S. lycopersicon*	20/20	15–21	+	+	C, N
	*A. conyzoides*	2/2	15–18	+	+	CR, LC
	*S. oleraceus*	0/5	-	-	-	-
AEV^SOL^	*N. benthamiana*	22/30	12–18	+	-	-
AYLCB^SOL^	*N. benthamiana*	00/30	-	-	-	-
pGreen0029	*N. benthamiana*	00/20	-	-	-	-
AEV^ACL^ and AYLCB^ACL^	*N. benthamiana*	10/10	12–18	+	+	R, LC, C
	*S. lycopersicon*	10/10	15–21	+	+	SG, C, R, N
	*A. conyzoides*	2/2	15–18	+	+	CR, LC
	*S. oleraceus*	0/5	-	-	-	-
none	*N. benthamiana*	00/20	-	-	-	-
pGreen0029	*N. benthamiana*	00/20	-	-	-	-

^#^ Days between inoculation and the first appearance of symptoms. * Symptoms exhibited by inoculated plants. Symptoms are denoted as vein yellowing (VY), leaf curl (LC), leaf crumpling (CR), stunted growth (SG), dimples on upper leaf surface (D), chlorosis (C), necrosis (N) reduced leaf area (R).

The inoculations of AEV^SOL^ alone, AYLCB^SOL^ alone, and pGreen vector alone, in *N. benthamina* did not show any apparent symptom (Table 2). None of the symptoms was observed in healthy control *N. benthamiana* plants. The *N. benthamiana* plants, inoculated with AEV^SOL^ and AYLCB^SOL^, produced dimples, leaf curl and chlorosis symptoms. A fragment of the expected size was obtained from *N. benthamiana* plants inoculated with AEV^SOL^ alone in a PCR. The PCR product was sequenced and showed 100% nucleotide sequence identity to the inoculated AEV. The results showed that AEV^SOL^ alone is infectious but does not induce symptoms.

All attempts to infect *S. oleraceus* with AEV and AYLCB were unsuccessful. This was likely due to the plants exuding a white latex following inoculation by the pin-prick method used here, meaning that the *Agrobacterium* inoculum was likely not retained by the plant long enough for T-DNA transfer and establishment of an infection.

## 4. Discussion

The begomovirus species AEV was first established in 2003, when the presently applicable species demarcation criteria for the genus *Begomovirus* were established, based upon the sequence of the Nepal isolate (AJ437618), which is the type isolate of the species [30]. Since this time AEV has been shown to be widespread across northern India and to infect a number of distinct hosts [31,32,33,34,35].

The results obtained here, as well as the results of Kumar *et al.* [31] and Marwal *et al.* [36], suggest that the common partner of AEV is the betasatellite species AYLCB. For none of the other AEV sequences available in the databases has the associated betasatellite been identified. Of the other AYLCB isolates available in the databases, only for two have the helper begomovirus been identified –*Tobacco curly shoot virus* isolated from wild sunflower originating from India (for HQ407397) and *Alternanthera yellow vein virus* (AlYVV) in *Sonchus arvensis* originating from Pakistan (for FN432358) [37]. It is interesting to note that the AYLCB from *S. arvensis* was identified in co-infection with a second betasatellite, Cotton leaf curl Multan betasatellite (CLCuMB). The identification of AYLCB with several distinct begomoviruses suggests that, as has been shown for other betasatellites [9,10,35,38], this has the capacity to be trans-replicated and maintained by more than one begomovirus.

The results presented here demonstrated the infectivity of two of the AEV clones and their respective AYLCB clones. Both pairs of clones were highly infectious to a range of plants and, for AEV^ACL^/AYLCB^ACL^, infectivity was shown to the plant species from which the clones were isolated (*A. conyzoides*). However, the symptoms (crumpling and curling of leaves, with no evidence of yellowing) induced by the cloned virus/betasatellite in *A. conyzoides* did not resemble the symptoms exhibited by the plant from which the clones were obtained (vein yellowing). The reason for this is unclear. Possibly the plant contained additional viruses/betasatellites that were responsible for the yellow vein symptoms, although there was no evidence for this. Nevertheless, Koch’s postulates for this isolate inducing yellow vein disease of *Ageratum* have not been satisfied. Possibly the *Ageratum* plant from which AYLCB^ACL^ was isolated contained more than one “type” of AYLCB. Certainly this betasatellite species is associated with, and thus likely can induce, a range of distinct symptoms. Kumar *et al.* [31] showed that an AEV isolate from carrot, with an AYLCB from *A. conyzoides*, induced typical yellow vein symptoms in *A. conyzoides.* AYLCB clones have also previously been shown to be infectious in the presence of *Ageratum yellow vein virus*, a virus that is not reported to be associated with this species of betasatellite, inducing typical yellow vein symptoms in *A. conyzoides* [35].

The “dimple” symptom, exhibited by the *A. conyzoides* plant from which AEV^ACN^ was isolated and induced by AEV^SOL^/AYLCB^SOL^ infection of *N. benthamiana* and *N. tabacum* are unusual. This symptom, as far as we are aware, has not been previously reported for any begomovirus infection of plants and was not noted by Kumar *et al.* [31] for their inoculations of AEV and AYLCB. On the underside of the leaf these structures look much like the enations on veins typical of cotton leaf curl disease due to hypo- and hyperplasia of spongy parenchyma induced by the βC1 protein of CLCuMB [39]. However, whereas for CLCuMB there are no changes on the upper leaf surface, here there is a depression (dimple) of the vein, indicating that distinct anatomical changes are occurring. Microscopic analysis will be required to determine what histological changes are occurring. Amin *et al.* [40] have shown that expression of the AC4 (a homolog of the C4 proteins of monopartite begomoviruses) protein of *Cabbage leaf curl virus* (a bipartite begomovirus originating from the New World which is only distantly related to AEV) from a *Potato virus X* vector induced a very similar dimple structure on leaves of *N. benthamiana.* For AEV however, infection of *N. benthamiana* in the absence of the betasatellite did not induce symptoms, suggesting that in this case the unusual symptom is due betasatellite rather than the virus.

It is evident, both from the results presented here and from the work of others, that AEV has a large natural host range that includes species from the families *Brassicaceae* (turnip), *Asteraceae* (*A. conyzoides*, *Z. elegans*, *C. crepidioides*, *Tagetes patula* and *S. oleraceous*), *Amaranthaceae* (*A. cruentus*), *Cucurbitaceae* (*T. dioica*), *Apiaceae* (carrot) and *Cleomaceae* (*C. gynandra*). This indicates that AEV is predominantly a weed infecting begomovirus, although it apparently has the capacity to occasionally infect crop species and is reported to be causing losses of the minor grain crop *A. cruentus* [34] and carrot [31].

The availability of infectious AEV and AYLCB clones that induce distinct symptoms in common hosts opens up the possibility of investigating the molecular basis for these differences by, for example, exchanging betasatellite components. It will be particularly interesting to discover the basis for the unusual symptoms (dimples), which have not been reported for any other begomovirus. This will be the focus of our future studies.

## Supplementary Files

Supplementary File 1
