# Supplementary material for: Ageratum enation virus—A Begomovirus of Weeds with the Potential to Infect Crops
Source: Viruses. 2015 Feb 10;7(2):647–65. doi: 10.3390/v7020647 (PMC4353908; doi:10.3390/v7020647)
Supplement: Supplementary File 1 [file viruses-07-00647-s001.pdf]

Supplementary Information

Ageratum enation virus—A Begomovirus of Weeds with the Potential to Infect Crops

Muhammad Tahir, Imran Amin, Muhammad Saleem Haider, Shahid Mansoor and Rob W. Briddon

Supplementary Table 1. Nucleotide sequence identities between the complete nucleotide sequences of the begomovirus (isolates SOL, ACL, ABF and ACN) and other selected begomoviruses available in the databases.

Isolates	RaLCV (3) *	PedLCV (5) *	PaLCuV (6) *	TbCSV (7) *	AEV (26) *	ABF	ACN	ACL	SOL
SOL	81.0–83.6	81.1–83.1	74.3–80.7	83.4–86.0	91.3–98.7	95.2	95.2	98.7	100
ACL	80.9–83.5	81.2–83.0	74.0–80.6	83.2–85.7	91.1–98.7	95.6	95.2	100	
ACN	81.5–83.5	81.5–83.6	74.3–81.0	82.6–85.6	88.9–98.0	97.8	100		
ABF	81.9–84.1	81.6–83.9	74.9–81.5	82.9–85.6	88.9–97.8	100			
AEV (26) *	81.1–87.3	81.6–86.5	74.1–81.4	82.8–87.4	89.1–100				
TbCSV (7) *	80.0–83.9	78.7–82.2	71.7–79.2	91.5–99.7					
PaLCuV (6) *	74.6–83.5	76.1–87.5	87.6–100						
PedLCV (5) *	83.1–90.5	88.6–96.8							
RaLCV (3) *	96.3–99.0								

* The figures in brackets indicate the number of sequences of a particular virus species in the databases which were used for the comparisons.
